# Supplementary material for: Metformin improves cognition of aged mice by promoting cerebral angiogenesis and neurogenesis
Source: Aging (Albany NY). 2020 Sep 16;12(18):17845–62. doi: 10.18632/aging.103693 (PMC7585073; doi:10.18632/aging.103693)
Supplement: Supplementary Figures [file aging-12-103693-s001..pdf]

SUPPLEMENTARY FIGURES

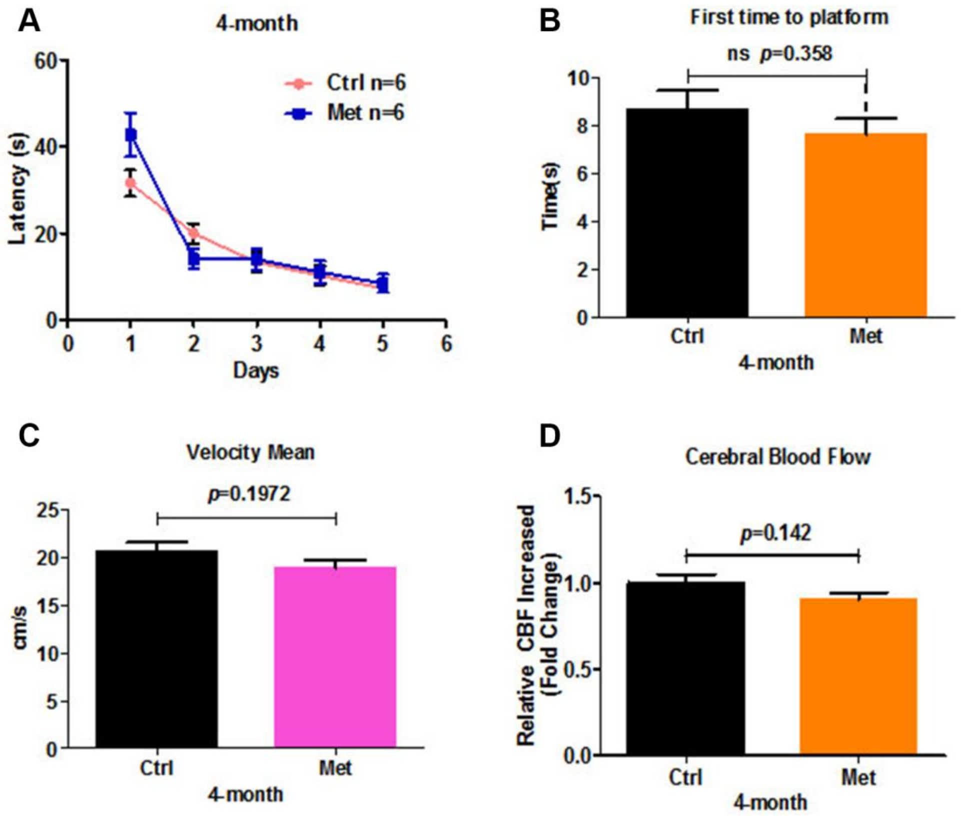

Supplementary Figure 1. Metformin did not improve spatial learning and memory of 4-month-old mice in the Morris water maze test. (**A**) Mean escape latency during 5 training days was not different in 4-month-old mice treated with metformin (n = 5) compared with the control group (n = 6). (**B**) Probe tests conducted 24 h after the acquisition phase indicated that the first time -to-platform was no different in 4-month-old mice treated with metformin than the control group. (**C**) Mean speed was also not different between the two groups. (**D**) Fold-change relative to the control group (n = 6) in cerebral blood flow (CBF) response to contralateral whisker stimulation was no difference in 4-month-old mice treated with metformin (n = 5) Ctrl: Control; Met: Metformin. The overall significance between two groups was determined by Student's *t*-test. * $p < 0.05$, ** $p < 0.01$, *** $p < 0.001$, ns, not significant.

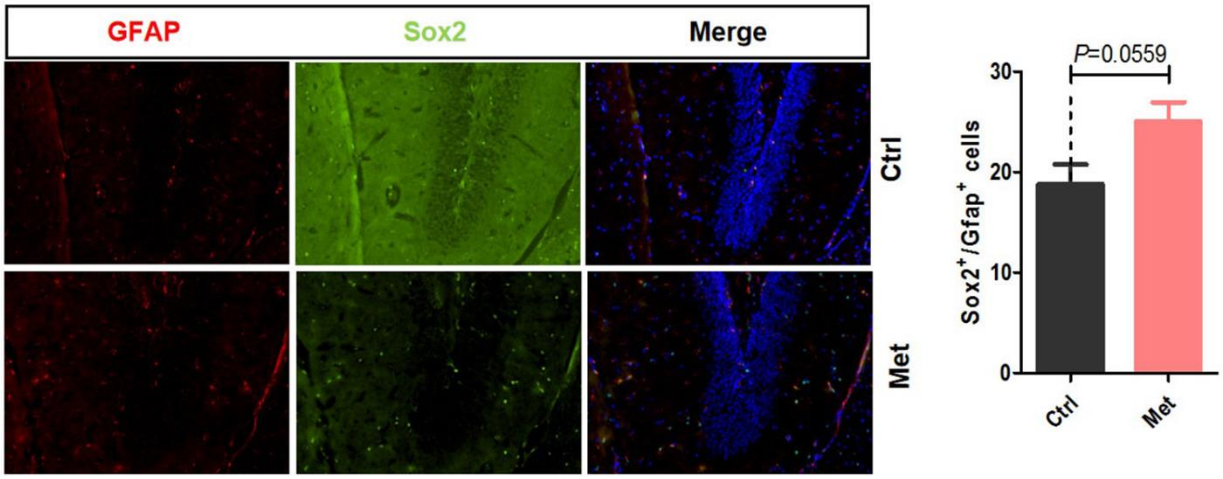

Supplementary Figure 2. DG of old mice treated with metformin (n ≥ 3) immunostained with the neural stem cell marker Sox2 and newborn neurons marker GFAP. Right: Quantification of Sox2+ /Gfap+ cells.

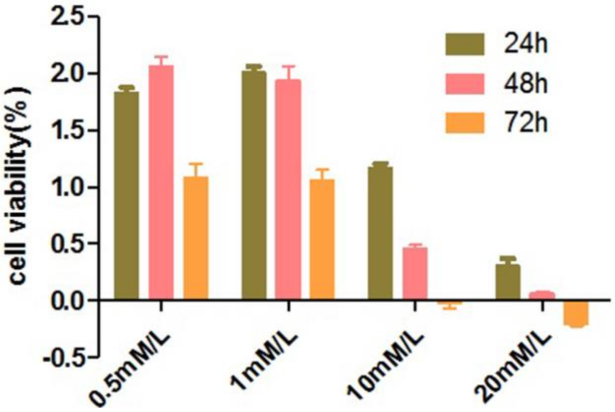

Supplementary Figure 3. Cell proliferation status analysis. NSCs were exposed to different concentrations of metformin (0.5mM/L, 1mM/L, 10mM/L, 20mM/L) for 24 h, 48 h and 72 h followed CCK8 assay.

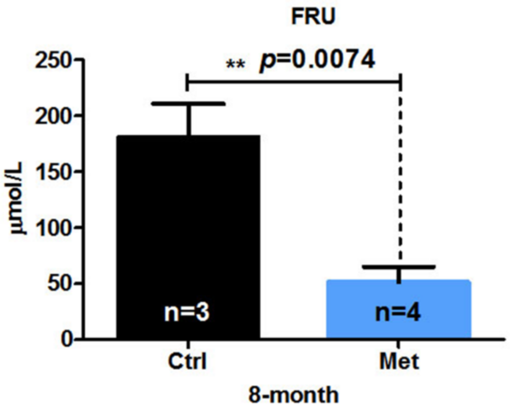

Supplementary Figure 4. Concentration of plasma fructosamine was decreased in 8-month-old mice treated with metformin (*n* = 4) compared with controls (*n* = 3). Ctrl: Control; Met: Metformin; FRU: Fructosamine. The overall significance between two groups was determined by Student's *t*-test. * $p < 0.05$, ** $p < 0.01$.

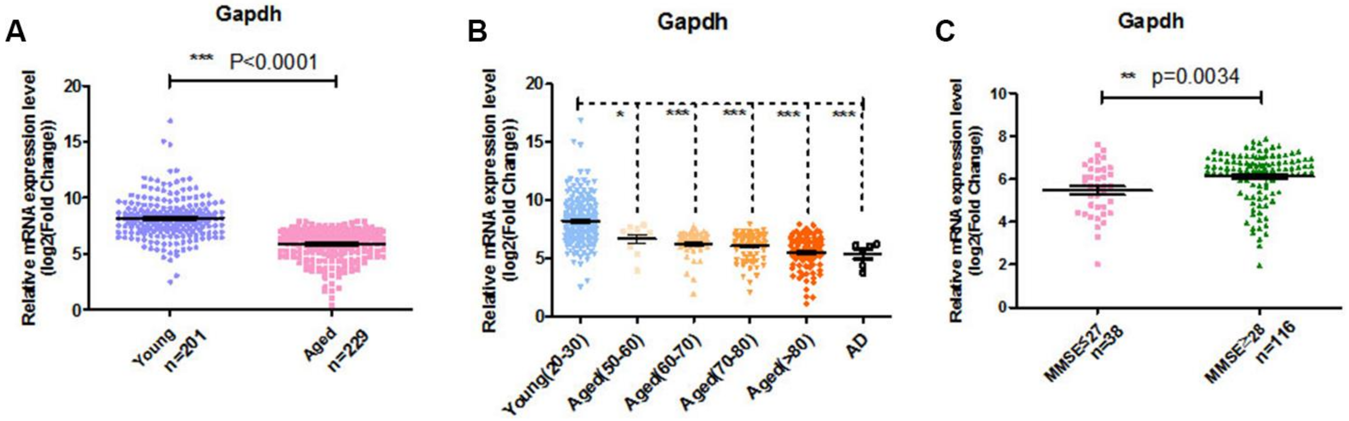

Supplementary Figure 5. GAPDH levels positively correlate with cognitive levels. in humans. (**A**) Relative GAPDH mRNA expression in blood of young and aged people. (**B**) Relative GAPDH mRNA expression in blood sample from people of various ages. (**C**) Relative GAPDH mRNA expression in blood sample of old people (chronicle age > 70s) positively correlated with cognitive levels measured by the Mini-Mental State Examination. * $p < 0.05$, ** $p < 0.01$, *** $p < 0.001$, ns, not significant.
